# Supplementary material for: Immune mediation of HMG-like DSP1 via Toll-Spätzle pathway and its specific inhibition by salicylic acid analogs
Source: PLoS Pathog. 2021 Mar 25;17(3):e1009467. doi: 10.1371/journal.ppat.1009467 (PMC8023496; doi:10.1371/journal.ppat.1009467)
Supplement: S1 Table — (DOCX) [file ppat.1009467.s001.docx]

**S1 Table. Primers used in this study**

| **Genes/Location** | **Primer sequences** | **Amplicon size** |
| --- | --- | --- |
| Se-Toll1 | 5´- CGCACTATGGGAACTCTCTT -3´  5´- CGTAGAGAAGACACCGTTGA -3´ | 298 bp |
| Se-Toll2 | 5´- TCCCAACAGCGTGGAGTTAC-3´  5´- ATGGAACGTGTGTTACGCCT-3´ | 335 bp |
| Se-Toll3 | 5´- GTGGAAACCCGTTTCAGTGC-3´  5´- GCGTCCATAGGTATGCTGCT-3´ | 355 bp |
| Se-Toll4 | 5´- CAACGTACGGACACTGTCAT-3´  5´- CTCGTTGTTCATGGAGCGTA-3´ | 247 bp |
| Se-Toll5 | 5´-TGGGTGACTGAAGAACTTGC-3´  5´-TACCTGATGGAGAGCCGATT-3´ | 207 bp |
| Se-Toll6 | 5´- CAAGTAAAGACCGACGGAGG-3´  5´- GTGGTCAGGTACTTGCTCTG-3´ | 362 bp |
| Se-Toll7 | 5´- GTATGCAGCATGGAGTTGGA -3´  5´- CAATCCGTGTTGTGTGTTCG -3´ | 423 bp |
| Se-Toll8 | 5´- ATGGAGCTTTTGCTGAGCTT -3´  5´- ACGCTCGTGATCTGATTGTC -3´ | 352 bp |
| Se-Toll9 | 5´- ACAACTTGACATGAACGCCT -3´  5´- CGGACAGAGAAAGTCGGAAG -3´ | 253 bp |
| Se-Toll10 | 5´- ACAGCGAAATGTTGAAAGCG -3´  5´- CGGGAACGATGAGTGTATCC -3´ | 213 bp |
| Se-Spz1 | 5´- TCGTCCAAAACCAGTACAGC-3´  5´- TCCCTCCCGACAGTACATTT-3´ | 220 bp |
| Se-Spz2 | 5´-CGACGAAAACGAAACGACTG-3´  5´-TCGTACTGTCCCGCTTCTAT-3´ | 232 bp |
| RL32 | 5´- ATGCCCAACATTGGTTACGG-3´  5´- TTCGTTCTCCTGGCTGCGGA-3´ | 270 bp |
| Gallerimycin | 5´-TCA GTC ATG AAA GCT TGC GTA-3´  5´-TCG CAC ACA TTG GCA TCC ATT C-3´ | 250 bp |
| Gloverin | 5´-CGT GGA CAT CTT CAG GGC C-3´  5´-GTC GTG TTC AAT GCC ACC-3´ | 275 bp |
| Lysozyme | 5´-ATG CAA AAG CTA ACG GTT TTC-3´  5´-GAT TCT TCC ATC CAT ACC AG-3´ | 242 bp |
| CRISPR mutant | 5’-CCC TAG AAG ATG GTG CCT T -3’  5’- CCT GGT GCA ATG TGC TTA AT -3’ | 808 bp |
