# Supplementary material for: Immune mediation of HMG-like DSP1 via Toll-Spätzle pathway and its specific inhibition by salicylic acid analogs
Source: PLoS Pathog. 2021 Mar 25;17(3):e1009467. doi: 10.1371/journal.ppat.1009467 (PMC8023496; doi:10.1371/journal.ppat.1009467)
Supplement: S2 Table — (DOCX) [file ppat.1009467.s002.docx]

**S2 Table.** **GenBank accession numbers used for phylogenetic analysis**

| **Species** | **Gene** |  | **Acronym** | **Accession number** | |
| --- | --- | --- | --- | --- | --- |
| *Drosophila melanogaster* | Spatzle1 |  | Dm-Spz1 | | NM_079802 |
|  | Spazle2 |  | Dm-Spz2 | | NM_168090 |
|  | Spazle3 |  | Dm-Spz3 | | AAF52574.2 |
|  | Spatzle4 |  | Dm-Spz4 | | AAF53100.2 |
|  | Spatzle5 |  | Dm-Spz5 | | NP_647753.1 |
|  | Spatzle6 |  | Dm-Spz6 | | NP_611961.1 |
| *Aedes aegypti* | Spatzle1 |  | Aa-Spz1 | | XM_001663577 |
|  | Spatzle2 |  | Aa-Spz2 | | XM_001659134 |
|  | Spatzle3 |  | Aa-Spz3 | | XM_001653272 |
|  | Spatzle4 |  | Aa-Spz4 | | EF173379 |
|  | Spatzle5 |  | Aa-Spz5 | | XM_001654288 |
|  | Spatzle6 |  | Aa-Spz6 | | XM_001655868 |
| *Bombyx mori* | Spatzle1 |  | Bm-Spz1 | | NP_001108066.1 |
|  | Spatzle4 |  | Bm-Spz4 | | AXJ14419.1 |
|  | Spatzle5 |  | Bm-Spz5 | | AMR08002.1 |
| *Spodoptera exigua* | Spatzle1 |  | Se-Spz1 | | MW286448 |
|  | Spatzle2 |  | Se-Spz2 | | MW286449 |
| *Drosophila melanogaster* | Toll1 |  | Dm-Toll1 | | AAA28941.1 |
|  | Toll2 |  | Dm-Toll2 | | AAF57509.1 |
|  | Toll3 |  | Dm-Toll3 | | AAF54021.3 |
|  | Toll4 |  | Dm-Toll4 | | AAF52747.3 |
|  | Toll5 |  | Dm-Toll5 | | AAF53306.1 |
|  | Toll6 |  | Dm-Toll6 | | AAF49645.1 |
|  | Toll7 |  | Dm-Toll7 | | AAF57514.1 |
|  | Toll8 |  | Dm-Toll8 | | AAF49650.1 |
|  | Toll9 |  | Dm-Toll9 | | AAF51518.2 |
| *Bombyx mori* | Toll1 |  | Bm-Toll1 | | Cheng et al. (2008) |
|  | Toll2 |  | Bm-Toll2 | |  |
|  | Toll3 |  | Bm-Toll3 | |  |
|  | Toll4 |  | Bm-Toll4 | |  |
|  | Toll5 |  | Bm-Toll5 | |  |
|  | Toll6 |  | Bm-Toll6 | |  |
|  | Toll7 |  | Bm-Toll7 | |  |
|  | Toll8 |  | Bm-Toll8 | |  |
|  | Toll9 |  | Bm-Toll9 | |  |
|  | Toll10 |  | Bm-Toll10 | |  |
|  | Toll11 |  | Bm-Toll11 | |  |
|  | Toll12 |  | Bm-Toll12 | |  |
|  | Toll13 |  | Bm-Toll13 | |  |
| *Spodoptera exigua* | Toll1 |  | Se-Toll1 | | MW286450 |
|  | Toll2 |  | Se-Toll2 | | MW286451 |
|  | Toll3 |  | Se-Toll3 | | MW286452 |
|  | Toll4 |  | Se-Toll4 | | MW286453 |
|  | Toll5 |  | Se-Toll5 | | MW286454 |
|  | Toll6 |  | Se-Toll6 | | MW286455 |
|  | Toll7 |  | Se-Toll7 | | MW286456 |
|  | Toll8 |  | Se-Toll8 | | MW286457 |
|  | Toll9 |  | Se-Toll9 | | MW286458 |
|  | Toll10 |  | Se-Toll10 | | MW286459 |
