# Supplementary material for: Immune mediation of HMG-like DSP1 via Toll-Spätzle pathway and its specific inhibition by salicylic acid analogs
Source: PLoS Pathog. 2021 Mar 25;17(3):e1009467. doi: 10.1371/journal.ppat.1009467 (PMC8023496; doi:10.1371/journal.ppat.1009467)
Supplement: S1 Fig — (A) Egg developmental stage. (B) Larval hemocytes. (C) Larval midgut. A ribosomal RNA, RL32, was used as endogenous control. Expression level was calculated as fold change from the lowest expression value. Each treatment was independently replicated three times. (DOCX) [file ppat.1009467.s003.docx]

**A**

**B C**
