# Supplementary figures and images for: Immune mediation of HMG-like DSP1 via Toll-Spätzle pathway and its specific inhibition by salicylic acid analogs
Source: PLoS Pathog. 2021 Mar 25;17(3):e1009467. doi: 10.1371/journal.ppat.1009467 (PMC8023496; doi:10.1371/journal.ppat.1009467)

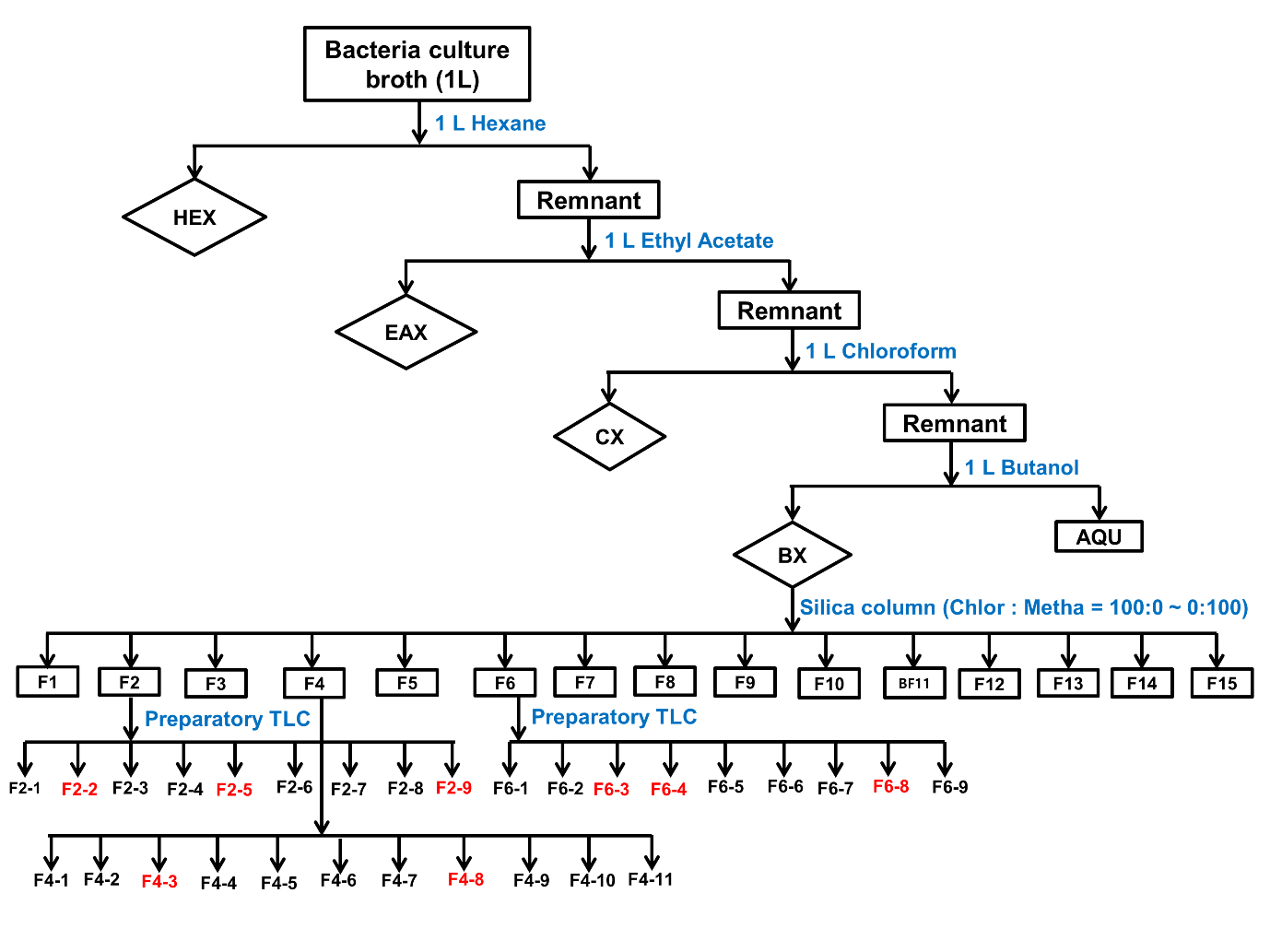

Supplement: S4 Fig — Organic extracts were obtained using hexane (‘HEX’), ethyl acetate (‘EAX’), chloroform (‘CX’), and butanol (‘BX’). BX was fractionated using a chromatography column filled with silica gel where a gradient chloroform/methanol mixture with increasing amount of methanol from 100:0 to 0:100 (v/v) was used. Active butanol fractions were separated using a preparatory thin layer chromatography (‘TLC’). (DOCX) [file ppat.1009467.s006.docx]

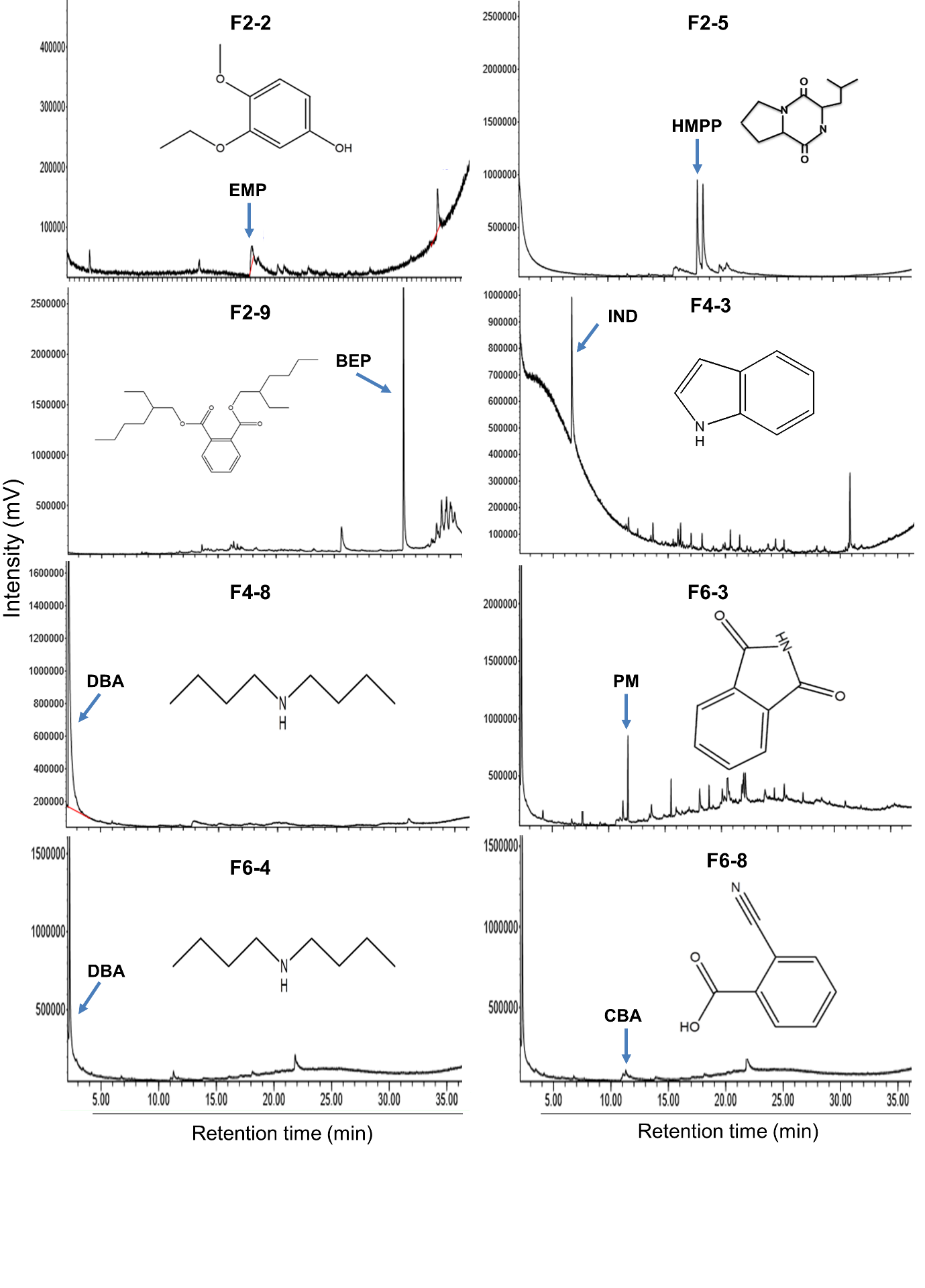

Supplement: S5 Fig — EMP, 3-ethoxy-4-methoxy phenol; HMPP, hexahydro-3-(2-methylpropyl)-pyrrolo[1,2-a]pyrazine-1,4-dione; BEP, bis (2-ethylhexyl) phthalate; IND, indole; DBA, dibutylamine; PM, phthalimide; CBA, o-cyanobenzoic acid. (DOCX) [file ppat.1009467.s007.docx]
