# Supplementary material for: Immune mediation of HMG-like DSP1 via Toll-Spätzle pathway and its specific inhibition by salicylic acid analogs
Source: PLoS Pathog. 2021 Mar 25;17(3):e1009467. doi: 10.1371/journal.ppat.1009467 (PMC8023496; doi:10.1371/journal.ppat.1009467)
Supplement: Supplementary file 5 [file ppat.1009467.s004.docx]

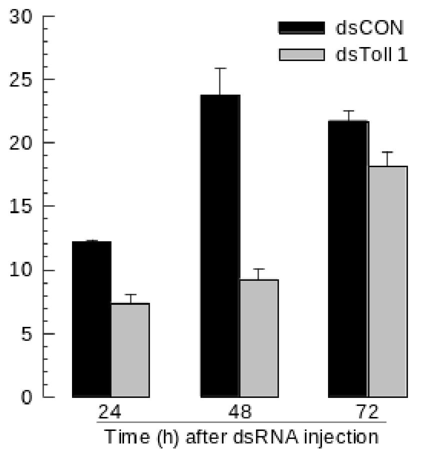

30
25
20
15
10
5
0
dsCON
dsToll 1
24
48
72
Time (h) after dsRNA injection

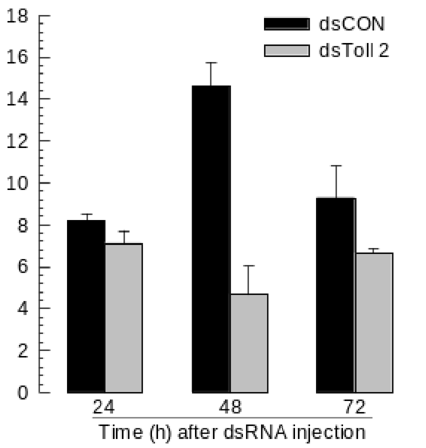

18
16
14
12
10
8
6
4
2
0
dsCON
dsToll 2
24
48
72
Time (h) after dsRNA injection

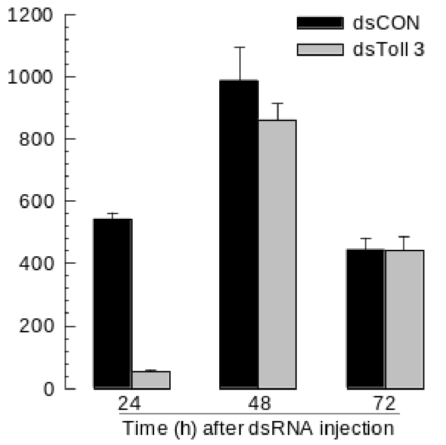

1200
1000
800
600
400
200
0
dsCON
dsToll 3
24
48
72
Time (h) after dsRNA injection

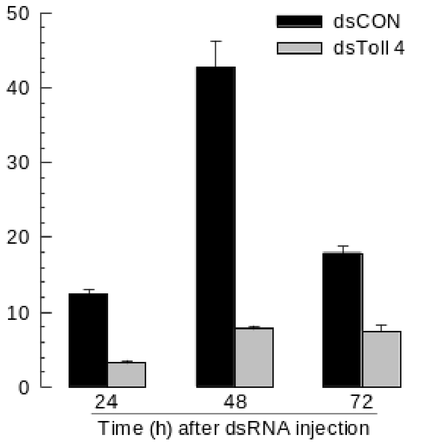

50
40
30
20
10
0
dsCON
dsToll 4
24
48
72
Time (h) after dsRNA injection

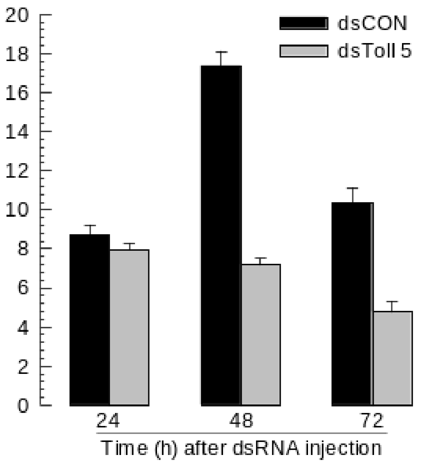

20
18
16
14
12
10
8
6
4
2
0
dsCON
dsToll 5
24
48
72
Time (h) after dsRNA injection

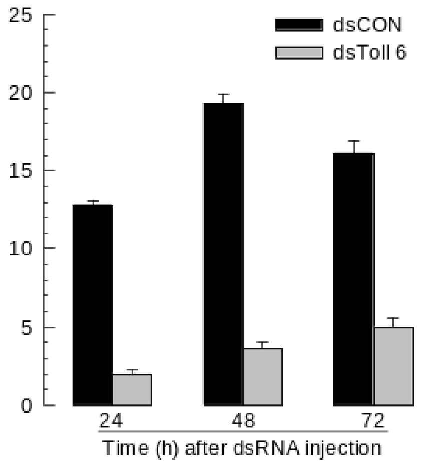

25
20
15
10
5
0
dsCON
dsToll 6
24
48
72
Time (h) after dsRNA injection

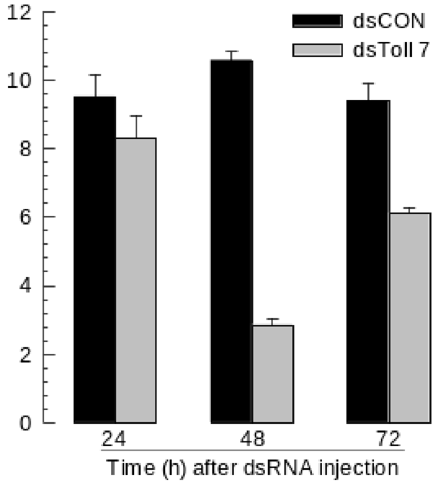
dsCON
dsToll 7
0
2
4
6
8
10
12
24
48
72
Time (h) after dsRNA injection

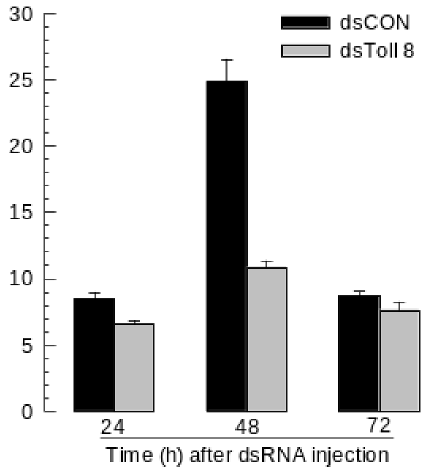
dsCON
dsToll 8
0
5
10
15
20
25
30
24
48
72
Time (h) after dsRNA injection

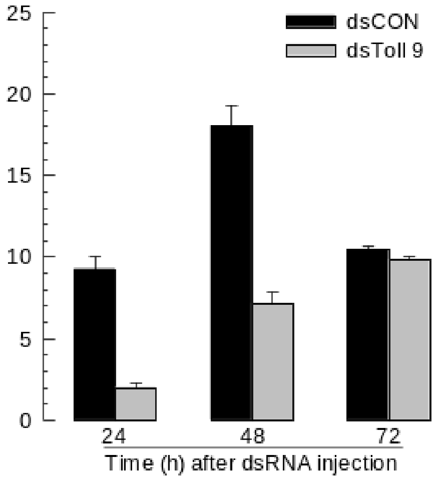
dsCON
dsToll 9
0
5
10
15
20
25
24
48
72
Time (h) after dsRNA injection

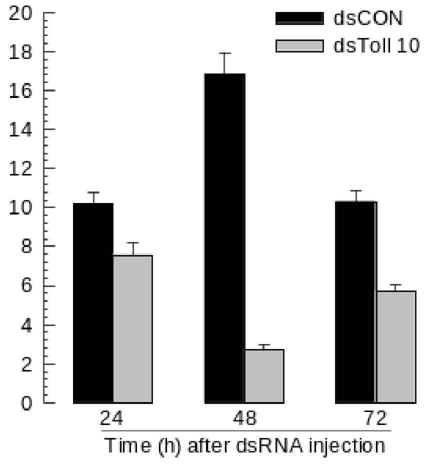
dsCON
dsToll 10
0
2
4
6
8
10
12
14
16
18
20
24
48
72
Time (h) after dsRNA injection
