# Supplementary material for: Immune mediation of HMG-like DSP1 via Toll-Spätzle pathway and its specific inhibition by salicylic acid analogs
Source: PLoS Pathog. 2021 Mar 25;17(3):e1009467. doi: 10.1371/journal.ppat.1009467 (PMC8023496; doi:10.1371/journal.ppat.1009467)
Supplement: Supplementary file 6 [file ppat.1009467.s005.docx]

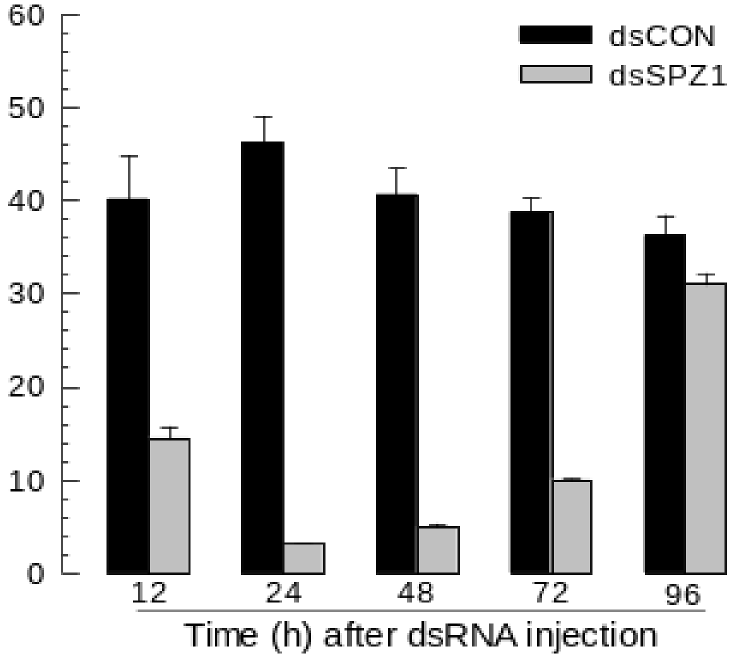
60
50
40
30
20
10
0
dsCON
dsSPZ1
12
24
48
72
96
Time (h) after dsRNA injection

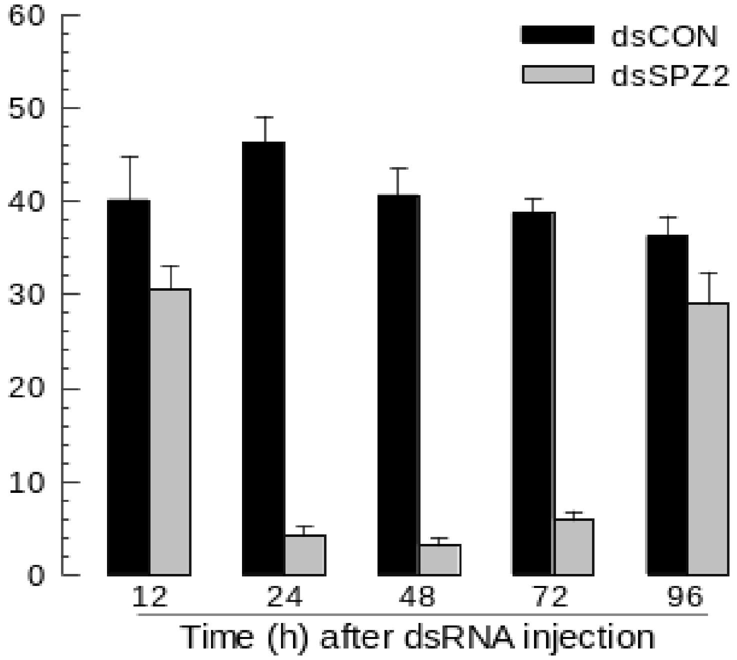
60
50
40
30
20
10
0
dsCON
dsSPZ2
12
24
48
72
96
Time (h) after dsRNA injection
