# Supplementary material for: Immune mediation of HMG-like DSP1 via Toll-Spätzle pathway and its specific inhibition by salicylic acid analogs
Source: PLoS Pathog. 2021 Mar 25;17(3):e1009467. doi: 10.1371/journal.ppat.1009467 (PMC8023496; doi:10.1371/journal.ppat.1009467)
Supplement: Attachment — Submitted filename: Response to reviewers.docx [file ppat.1009467.s008.docx]

**Response to reviewers’ comments**

**[Reviewer #1]**

**Comment #1-1**: Abstract: The abstract is incredibly long and it seems like much of this information belongs in the Introduction. Further, from the abstract, the reader expected eicosanoid biosynthesis to be studied in the manuscript and it was not.

**Response**: Abstract is reduced by deleting introductory explanation from line 24 to line 30.

**Comment #1-2**: Line 104 – explain to the reader what Pelle kinase is.

**Response**: It is added as follows: “Toll immune signalling is likely to serve as a functional link between DSP1 and PLA2 activation because Pelle kinase, an adaptor molecule of Toll receptor, is required for PLA2 activation in S. exigua upon bacterial challenge [17].”

**Comment #1-3**: Paragraph starting at line 107 – it is not clear what the authors think the order of events is.

**Response**: It is re-written as follows: “This led us to pose a hypothesis that DSP1 acts as an initial immune inducer by activating the Toll-Spätzle signal pathway to activate PLA2.”

**Comment #1-4**: Figure 1 – The change in Se-DSP1 immunofluorescence isn’t convincing. It seems like it is relocalizes, perhaps to the nucleolus, but the intensity does not appear reduced. Some quantification is needed to clarify this.

**Response**: The amount of DSP1 is estimated by measuring intensity. The information is added to the result.

| Treatment | % Fluorescence Intensity for Se-DSP1 (relative to Naive) | | | | | |
| --- | --- | --- | --- | --- | --- | --- |
|  | Replication 1 | Replication 2 | Replication 3 | Mean | Std dev | Group |
| Naive | 100 | 86.16 | 92.25 | 92.80333 | 6.936572 | A |
| *E. mundtii* | 59.57 | 47.06 | 63.49 | 56.70667 | 8.581097 | B |

Statistical analysis: t = 5.67, df = 4, P = 0.0048

**Comment #1-5**: The terminology in the results and in the figures is not always consistent. For example, Line 134-135, Se-DSP1 (rSe-DSP1) is not how it is written in the Figure 2. Another example is in relation to Figure 7

**Response**: All the recombinant protein information is written in the figure captions as follows:

Fig. 2. Immune mediation of Se-DSP1 in S. exigua. rSe-DSP1 (‘DSP1’) was injected into L5 larvae at a dose of 0.8 µg/larva. Inactivation of DSP1 used heat treatment at 95oC for 10 min. Immune challenge used an injection of E. mundtii (‘Em’) to L5 larvae at a dose of 4 × 105 cells/larva. At 8 h PI, hemolymph and fat body were collected. Hemolymph was used for analyzing activities of phenoloxidase (‘PO’) and sPLA2. Fat body was used for analyzing cPLA2 activity. For antimicrobial peptide (‘AMP’) analysis, fat body from treated larvae was collected at 12 h PI. (A) Up-regulation of PO activity by Se-DSP1. RNAi used injection of gene-specific dsRNA against Se-DSP1 (dsDSP1) at a dose of 1 µg/larva. At 24 h PI, Em was used for treatment. Control dsRNA (dsCON) used dsRNA specific to a viral gene, CpBV302. (B) Up-regulation of PLA2 activity by Se-DSP1. (C) Up-regulation of AMP gene expression. Expression levels of AMP were presented as fold changes in comparison with those in naïve larvae. Expression level of a ribosomal gene, RL32, was used as reference to normalize expression levels of target genes. Each treatment was replicated three times. Different letters above standard deviation bars denote significant difference among means at Type I error = 0.05 (LSD test).

Fig. 7. Deletion mutants of SeToll9 using CRISPR/Cas9 and their insensitivity to Se-DSP1 to express immune responses in S. exigua. (A) Construction of SeToll9-deletion mutants (ΔToll9) with two single-stranded guide RNAs (sgRNAs), with protospacer adjacent motif (PAM) denoted in red color. Twelve different types of ΔToll9 (‘M1-M12’) are confirmed by sequence analysis of 808 bp around both sgRNA-specific deletion sites by comparing with the corresponding sequence of wild type (‘Wild’). Deletion or insertion sizes by CRISPR/Cas9 are denoted by ‘+’ or ‘-’ in parentheses. (B) Insensitivity of mutants to Se-DSP1 in activation of PLA2. rSe-DSP1 (‘DSP1’) was injected into L5 larvae at a dose of 0.8 µg/larva. Control (‘CON’) larvae were injected with PBS. At 8 h PI, hemolymph and fat bodies were collected. Hemolymph was used for sPLA2 activity analysis. Fat body was used for cPLA2 activity analysis. (C) Insensitivity of mutants to Se-DSP1 in activating expression of antimicrobial peptide (gallerimycin (‘Gal’), gloverin (‘Glv’), and lysozyme (‘Lyz’)) genes. Fat bodies were collected from treated larvae at 12 h PI of Se-DSP1. Expression level of a ribosomal gene, RL32, was used as reference to normalize expression levels of target genes. Each treatment was replicated three times. Different letters above standard deviation bars denote significant difference among means at Type I error = 0.05 (LSD test).

**Comment #1-6**: The authors need use PLA2 activity to indicate eicosanoid biosynthesis, but PLA2 has many other roles. The authors need to discuss this limitation. Further, in the results it is not discussed what samples are used for the analysis and why they are the correct samples.

**Response**: We add the direct functional link in the discussion as follows: “Although PLA2 activity is required in various physiological processes, it is essential to mediate various immune responses in insects [4, 6].”

**Comment #1-7**: It seems it is worth discussing why AMP genes are activated by heat inactivated rSe-DSP1

**Response**: It might be a stress response to a physical damage. Thus we add the discussion as follows: “However, their up-regulated levels were much less than five-fold and regarded as a stress response to a physical damage by injection.”

**Comment #1-8**: The description of the data in Figure 4 is difficult to follow. Sometimes the authors say which Toll receptors have no effects and for other data which Toll receptors play a role. It would be clearer to the reader to talk about the Toll receptors that are involved in the process. Also a discussion of the difference in the Toll receptor role in PLA activity vs AMP gene expression is warranted. It is unclear what the reader should conclude about who is involved.

**Response**: In all immune responses mediated by Se-DSP1, only SeToll9 was commonly involved because its RNAi impaired the all responses. Thus we add the following sentence to make a conclusion. “From these analyses, only RNAi treatment against SeToll9 expression impaired all the immune responses mediated by Se-DSP1.”

**Comment #1-9**: Line 183 – the data in figure 5c would say that neither Spz gene is induced in the hemocytes in response to EM, and Spz1 may be inhibited. This is not what is stated in the results and it seems worth talking about. Lines 184-185 seems to contradict the prior sentence

**Response**: Yes, we agree on your interpretation. Even though Se-Spz1 mediates Se-DSP1 immune signaling to Toll receptor, its expression was not closely related with the immune challenge. To be clear, we add this interpretation to the text as follows: “In addition, rSe-DSP1 did not induce Se-Spz1 in fat bodies while it significantly induced the two Spz genes in hemocytes. These suggest that both Se-Spz expressions might be not directly induced by the bacterial challenge or Se-DSP1 injection.”

**Comment #1-10**: In the section starting at 209, it would help the reader for the authors to explain their method of assessing pathogenicity.

**Response**: We add the details to the paragraph as follows: “In addition, rSe-DSP1 did not induce Se-Spz1 in fat bodies while it significantly induced the two Spz genes in hemocytes. These suggest that both Se-Spz expressions might be not directly induced by the bacterial challenge or Se-DSP1 injection.”

**Comment #1-11**: Figure 1 legend talks about Tubulin western blots that are not shown in the figure

**Response**: Tubulin control is shown in Fig. 1C.

**Comment #1-12**: The RNAi knockdown data in Figure 4a and Figure6a are shown as connected lines. Such a graph implies the expression was assessed repeatedly in the same cells, when in reality it was done on different cells. The data should be presented as individual data points or bar graphs.

**Response**: We add the individual data to Supplementary data as follows:

**S2 Fig**

**S3 Fig**

**[Reviewer #2]**

**Comment #2-1**: This study showed the release of DSP1 from nucleus to plasma. The released DSP1 appears to be transported in plasma using exosome cargo. Here are questions. What does the intracellular signal stimulate DSP1 release from nucleus? Why does the DSP1 take exosome in the plasma?

**Response**: All the issues should be clarified in our subsequent studies. Here is our speculation at this moment. (1) DSP1 undergoes conformational change from oxidized to reduced form or vice versa. This conformational change may lead to translocation of DSP1 from nucleus. (2) DSP1 may have benefits from exosome cargo by protecting from degradation during transport and by specific targeting to immune-associated tissues.

**Comment #2-2**: In addition to Toll9, DSP1 may use Toll6, Toll7, or Toll8 to induce the AMP or PO activation from Fig. 4. Why did you determine Toll9 as DSP1 signal component?

**Response**: In all immune responses mediated by Se-DSP1, only SeToll9 was commonly involved because its RNAi impaired the all responses. Thus we add the following sentence to make a conclusion. “From these analyses, only RNAi treatment against SeToll9 expression impaired all the immune responses mediated by Se-DSP1.”

**Comment #2-3**: I am not sure that DSP1 directly activated Spz to trigger Toll signaling pathway?

**Response**: We do not think DSP1 bind to Spz to activate Toll. It may activate the upstream molecules such as serine proteases. This is because PO activation is mediated certain HP serine protease, which is at the signal upstream to Spz.

**Comment #2-4**: What is the relationship of Bt pathogenicity and immunosuppression? Is it required for the Bt to exhibit its insecticidal activity?

**Response**: Bt toxicity is derived from Crystal toxin, which form pores on midgut epithelium and lead to passage from lumen to hemocoel. This route is used by commensal microbes, which enter hemocoel to induce septicemia. Thus immune response is a defense to Bt pathogenicity. The immunosuppression is favored by Bt.

**Comment #2-5**: How can you explain the inhibitory activity of EMP on DSP1 release from the nucleus?

**Response**: We do not know from this study. We speculate that EMP binding to DSP1 prevent its conformational change, which prevent the DSP1 translocation from nucleus to hemcoel. This needs to be clarified in subsequent study as mentioned above.

**[Reviewer #3]**

**Comment #3-1**: Some insect pathogenic microbes overcome host immune responses to infection by secreting compounds that inhibit insect immune reactions to infection. Park and Kim (2000; doi: 10.1016/s0022-1910(00)00071-8) first reported that the bacterial lethality of the insect pathogen Xenorhabdus nematophilus was attenuated after injecting arachidonic acid (AA), a direct precursor to biosynthesis of prostaglandins and other eicosanoids, into the abdomens of infected lepidopterans, Spodoptera exigua. They put forth the idea the bacterium suppresses insect immunity by inhibiting biosynthesis of eicosanoids. Now, after 20 years and many publications on eicosanoid signaling in insect immunity, they turned attention to a related insect pathogen, X. hominickii. This pathogen also secretes metabolites that inhibit the first step in eicosanoid biosynthesis, phospholipase A2 (PLA2), which effectively suppresses eicosanoid signaling to suppress host immunity. They reported that a damage-associated molecular pattern called dorsal switch protein 1 (DSP1), which activates PLA2 early in infection in S. exigua. They used immunofluorescence to record increased hemocyte spreading and Se-DSP1 in fat body. They used western blots to show Se-DSP2 in hemolymph of infected, but not naïve larvae at 6 h post-infection. Their western blot analysis also indicated the Se-DSP1 was transported out the cells as via exosomes. The authors created a recombinant Se-DSP1 (rSe-DSP1) and used it to demonstrate that bacterial infection with another insect pathogen, E. mundtii, leads to increased PO activity, as does rDSP1 treatments, but not after denaturing the protein. Treating larvae with a dsRNA construct, dsDSP1 + E. mundtii blocked infection-triggered PO activity. They used enzyme activity assays to show rDSP1 treatments led to significant increases in secretory PLA2 and cellular PLA2 activities. Translating to the influence of rDSP1 on humoral immunity, the authors show that rDSP1 injections led to significant increases in mRNAs encoding a range of antimicrobial peptides and proteins, including apolipophoren-III, cecropin and lysozyme. They report ten Toll receptors and show accumulations of mRNAs encoding them. Expression of each of the ten genes was substantially reduced at 24 or 48 h after injecting dsRNA constructs specific to each gene. Co-injections with each dsRNA + rDSP1 led to substantial PO activity, sPLA2 activity and cPLA2 activity. Similar co-injections led to substantial expression of genes encoding three anti-microbial proteins. Digging deeper into humoral immunity, the authors report two genes encoding Spätzels, showing gene structures and a phylogenetic tree placing them withing Diptera-Lepidoptera. They show the genes are expressed in hemocytes and fat body. rDSP1 treatments led to increased gene expression in hemocytes, but not in fat body. Bacterial injections led to increased expression of both genes in fat body. They show dsRNA treatments led to decreased expression of both genes encoding Spätzes from 24 – 72 h post-injections. Bacterial injection and DSP1 injections led to increased PO activity, but not to increased PLA2 activity. Co-inections with dsSpätze1 + DSP1 did not lead to increased expression of three anti-microbial peptides, although dsSpätze2 +DSP1 did.

The authors continued drilling into humoral immune signaling by creating CRISPR/Cas9 deletion mutants in SeToll9. Although rDSP1 injections into wild-type larvae led to increased sPLA2 and cPLA2 activities and to increased expression of the three anti-microbial proteins, similar injections into the deletion mutants did not. The changes in immune parameters just mentioned translated into increased mortality following co-injections with most dsRNAs designed to each of the ten toll receptors + the insect pathogen, Bacillus turingiensis Mortality did not increases following dsTOLL1, 8 or 10. Similarly, dsSpätze1 and -2 treatments also led to increased mortality. The authors identified X. hominickii metabolites that bind to rSeDSP1, and thus, block its translocation. They used GC-MS analysis to identify several metabolites with binding affinities in the low μM range, of which 3-eethoxy-4-methoxyphenol (EMP) had the highest binding affinity. In a direct test of DSP1 translocation, the authors presented a western blot showing the presence of Se-DSP1 in plasma from larvae injected with the pathogen E. mundtii, but not in larvae injected with the pathogen + EMP. EMP + rDSP1 treatments led to sharp reductions in PO, sPLA2 and cPLA2 activities and virtually eliminated accumulations of mRNAs encoding gallerimycin, gloverin and lyzozyme. Injections of pathogen B. thuringiensis led to increased mortality in control larvae. The bacterial injections into larvae treated with dsDSP1 led to higher mortality and still higher mortality followed in larvae treated with EMP.

The authors complete their story with a meaningful model of a hypothesized mechanism of DSP1 action. In their view, the bacterial product EMP damages hemocytes and DSP1 is translocated from cells into hemolymph, where it activates one or more serine protease cascades that leads to active phenol oxidase and melanization reactions. It also activates two Spätzels, with interacts with toll receptors that lead to biosynthesis of eicosanoids that mediate cellular immune reactions and lead to expression of genes encoding lysozyme and other anti-microbial proteins. They present three supplementary figures. Fig. S1 reports accumulations a mRNAs encoding the 10 toll receptors. Fig. S2 shows their extraction scheme for analysis of the X. hominickii metabolites and Fig. S3 shows the chemical structures of selected metabolites.

The authors present a well-reasoned and thorough series of experiments to test their hypothesis.

**Response**: We appreciate your nice understanding on our findings and thoughtful comments.
