# Supplementary material for: Immune mediation of HMG-like DSP1 via Toll-Spätzle pathway and its specific inhibition by salicylic acid analogs
Source: PLoS Pathog. 2021 Mar 25;17(3):e1009467. doi: 10.1371/journal.ppat.1009467 (PMC8023496; doi:10.1371/journal.ppat.1009467)
Supplement: Attachment — Submitted filename: Response to reviewers-r2.docx [file ppat.1009467.s009.docx]

**Response to Editor’ comments**

**Comment**: I am happy with all the revisions in the main text, but would like you to look again at the first comment about the abstract. At the moment the abstract is hard to read unless you have technical knowledge of this area. This is because it includes a large amount of technical detail and goes through many different experiments. In contrast, it does not make clear the wider significance and importance of the work. This contrasts with the author summary, which did this job very well. I would ask that the abstract is simplified and makes clearer the wider significance of your work. I apologise for returning the manuscript a second time, but I hope this will be a quick change to make that will help your work reach a wider audience.

**Response**: To address this comments, we minimize the technical description while the objective and significance of the results are clearly explained. This effort resulted in reduction in the number of words from 275 to 223.

“Xenorhabdus hominickii, an entomopathogenic bacterium, inhibits eicosanoid biosynthesis of target insects to suppress their immune responses by inhibiting phospholipase A2 (PLA2) through binding to a damage-associated molecular pattern (DAMP) molecule called dorsal switch protein 1 (DSP1) from Spodoptera exigua, a lepidopteran insect. However, the signalling pathway between DSP1 and PLA2 remains unknown. The objective of this study was to determine whether DSP1 could activate Toll immune signalling pathway to activate PLA2 activation and whether X. hominickii metabolites could inhibit DSP1 to shutdown eicosanoid biosynthesis. Toll-Spätzle (Spz) signalling pathway includes two Spz (SeSpz1 and SeSpz2) and 10 Toll receptors (SeToll1-10) in S. exigua. Loss-of-function approach using RNA interference showed that SeSpz1 and SeToll9 played crucial roles in connecting DSP1 mediation to activate PLA2. Furthermore, a deletion mutant against SeToll9 using CRISPR/Cas9 abolished DSP1 mediation and induced significant immunosuppression. Organic extracts of X. hominickii culture broth could bind to DSP1 at a low micromolar range. Subsequent sequential fractionations along with binding assays led to the identification of seven potent compounds including 3-ethoxy-4-methoxyphenol (EMP). EMP could bind to DSP1 and prevent its translocation to plasma in response to bacterial challenge and suppress the up-regulation of PLA2 activity. These results suggest that X. hominickii inhibits DSP1 and prevents its DAMP role in activating Toll immune signalling pathway including PLA2 activation, leading to significant immunosuppression of target insects.”
